# Elevated temperature altered photosynthetic products in wheat seedlings and organic compounds and biological activity in rhizopshere soil under cadmium stress

**DOI:** 10.1038/srep14426

**Published:** 2015-09-23

**Authors:** Xia Jia, YongHua Zhao, WenKe Wang, Yunhua He

**Affiliations:** 1 School of Environmental Science and Engineering, Chang’an University, No. 126, Yanta Road, Xi’an 710054, People’s Republic of China; 2 Key Laboratory of Environmental Protection & Pollution and Remditation of Water and Soil of Shaanxi Province; 3 Key Laboratory of Subsurface Hydrology and Ecological Effect in Arid Region of Ministry of Education; 4 The School of Earth Science and Resources, Chang’an University, No. 126, Yanta Road, Xi’an 710054, People’s Republic of China

## Abstract

The objective of this study was to investigate the effects of slightly elevated atmospheric temperature in the spring on photosynthetic products in wheat seedlings and on organic compounds and biological activity in rhizosphere soil under cadmium (Cd) stress. Elevated temperature was associated with increased soluble sugars, reducing sugars, starch, and total sugars, and with decreased amino acids in wheat seedlings under Cd stress. Elevated temperature improved total soluble sugars, free amino acids, soluble phenolic acids, and organic acids in rhizosphere soil under Cd stress. The activity of amylase, phenol oxidase, invertase, β-glucosidase, and l-asparaginase in rhizosphere soil was significantly improved by elevated temperature under Cd stress; while cellulase, neutral phosphatase, and urease activity significantly decreased. Elevated temperature significantly improved bacteria, fungi, actinomycetes, and total microorganisms abundance and fluorescein diacetate activity under Cd stress. In conclusion, slightly elevated atmospheric temperature in the spring improved the carbohydrate levels in wheat seedlings and organic compounds and biological activity in rhizosphere soil under Cd stress in the short term. In addition, elevated atmospheric temperature in the spring stimulated available Cd by affecting pH, DOC, phenolic acids, and organic acids in rhizosphere soil, which resulted in the improvement of the Cd uptake by wheat seedlings.

The phenomenon of global warming has long been known to affect the physiology, development, growth, and productivity of plants^1^. Elevated atmospheric temperature has been the subject of many hypotheses and experiments centered around investigating the effect that elevated temperature has on the growth and development of plants. Elevated temperature can greatly hinder several stages of plant growth, particularly the flowering and fertilization stages resulting in overall decreased productivity during the summer season^2^,^3^. Increasing average temperature has been proven to lessen flowering time and decrease the total biomass and yield of wheat grain^4^. It is accepted knowledge that elevated temperature increases the rate of transpiration and stomatal conductance, while decreasing photosynthetic activity resulting in a significant reduction in the plant biomass yield^5^,^6^. However, studies on the effects of climate warming have indicated that the effects of elevated temperature on plants are positive, negative or neutral, depending on species and the period of exposure^7^,^8^,^9^. Modifications to products that result from the effect of elevated temperature on photosynthetic activity could potentially alter the microbial demand for nitrogen (N) and the flow of N between soil microorganisms and plant roots. Additionally, root exudation may react to elevated temperature in the same manner as photosynthetic activity and plant production^10^. Consequently, dissolved organic carbon (DOC) and organic compounds in soils may alter in relation to the shifts of root exudation which may affect soil biological activities and the survival of microorganisms. In summation, elevated atmospheric temperature causes an increase in soil enzymes, microbial activity, and soil nitrification in previous studies^11^,^12^.

Heavy metal contamination of soil is also a serious global problem. Among the heavy metals, cadmium (Cd) is widespread and is one of the most toxic pollutants of the surface soil layer. Cd is principally dispersed into agricultural soil by the utilization of phosphate fertilizers, application of sewage and industrial wastewater for irrigation, and atmospheric deposition from metallurgical industries (e.g. the incineration of plastics and batteries, and the burning of fossil fuels)^13^. Such pollution is becoming a serious environmental problem^14^. Many studies have focused on the effects of Cd contamination on crop growth, development, quality of plant, soil enzyme activity, and soil microorganisms^15^,^16^. Alkaline phosphomonoesterase, arylsulphatase, and protease activities and soil microbial biomass carbon all significantly decrease in Cd-contaminated soil^17^, and additional studies have shown that the concentration of root exudates increases significantly in Cd-contaminated soil^18^,^19^.

Although many studies have investigated the effect of temperature on plants^6^,^7^,^8^,^9^, few studies have dealt with the interactive effect of elevated atmospheric temperature and heavy metal contamination of soil on plants and soils. The response of photosynthetic products in plants to elevated temperature combined with Cd stress influences the release of organic compounds from the roots of plants into rhizosphere soil. It is estimated that up to 40% of the carbon fixed by plants can be lost through root exudation^20^. A wide variety of compounds released by plant roots can result in changes in DOC and organic compounds in rhizosphere soil and create unique microenvironments for soil microorganisms^21^,^22^, which in turn affects soil biological activity in the rhizosphere. The changes in DOC and organic compound concentrations in rhizosphere soil under elevated atmospheric temperature can affect soil pH and the bioavailability of Cd for plants and microorganisms. Additionally, changes in DOC and organic compounds in rhizosphere soil can influence the enzyme and microbial biological activities. It is well understood that soil biological activity plays an important role in soil fertility in these unique microenvironments.

For this reason, the aim of this study is to investigate the effects of slightly elevated atmospheric temperature in the spring on photosynthetic products in wheat seedlings and on organic compounds and biological activities in rhizosphere soil under Cd stress. Many studies have focused on the effects of either elevated atmospheric temperature or metal contamination on these variables, but little is known about their combined effects. We examined these effects on wheat seedlings, a crop that is one of the most widely studied crops^23^.

## Results

### Air temperature

**Figure 1:**
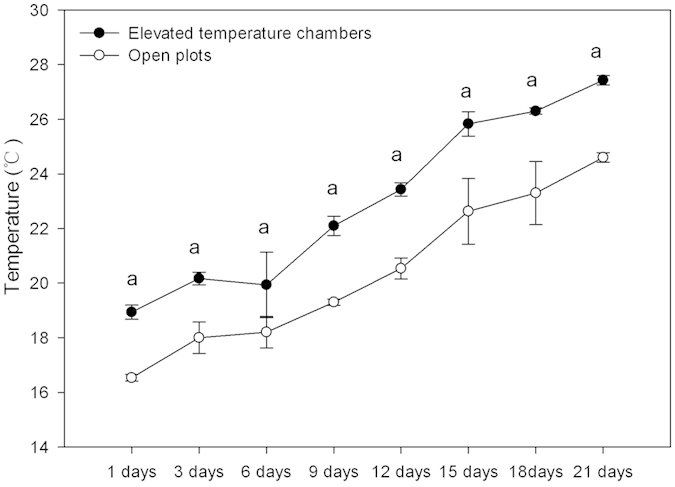
Mean daily air temperature (°C) in elevated temperature chambers and open plots. Data are means ± SE; (n = 3). Letter a stands for the difference between elevated temperature chambers and open plots at *p* < 0.01.

The air temperature averaged was 2.6 °C higher in the open-top chambers (OTCs) than in the open plots (Fig. 1). The temperature measured ranged from 18.9–23.4 °C and the difference between OTCs and open plots ranged from 1.7–2.9 °C during the first two weeks of the experiment, which indicated the elevated temperature was moderate for wheat seedlings rather than heat shock. However, air temperature measured was above 25 °C and the difference between OTCs and open plots ranged from 2.8–3.2 °C during the third week, which was beyond the optimal temperature of wheat growth. In summary, elevated temperature in this experiment was slight for wheat seedlings, which was defined as slightly elevated temperature by us.

### Concentration of available Cd in the soil before sowing and in rhizosphere soil at harvest

Under either open plots or elevated temperature, available Cd content in rhizosphere soil at harvest was lower than that in the soil before sowing, indicating that wheat seedlings was efficient in altering the bioavailability of Cd by affecting the pH value in rhizosphere soil (Table 1). Under three Cd levels (Cd0, Cd1, and Cd5), available Cd contents in rhizosphere soil under elevated temperature decreased significantly by 25.0%, 31.4%, 22.9%, respectively, compared with those under open plots. Furthermore, the difference of available Cd in between rhizosphere soil at harvest and soil before sowing was greater under the combined treatments than under open plots, which confirmed that elevated temperature enhanced available Cd in rhizosphere soil (Table 1).

### Total Cd uptake by wheat seedlings

Under three Cd levels (Cd0, Cd1, and Cd5), total Cd uptake by wheat seedlings under elevated temperature increased significantly by 15.5%, 22.8%, 28.4%, respectively, compared with that under open plots (Table 1), which showed that Cd uptake efficiency was enhanced by elevated temperature with increasing Cd levels.

### Photosynthetic products in wheat seedlings

**Figure 2:**
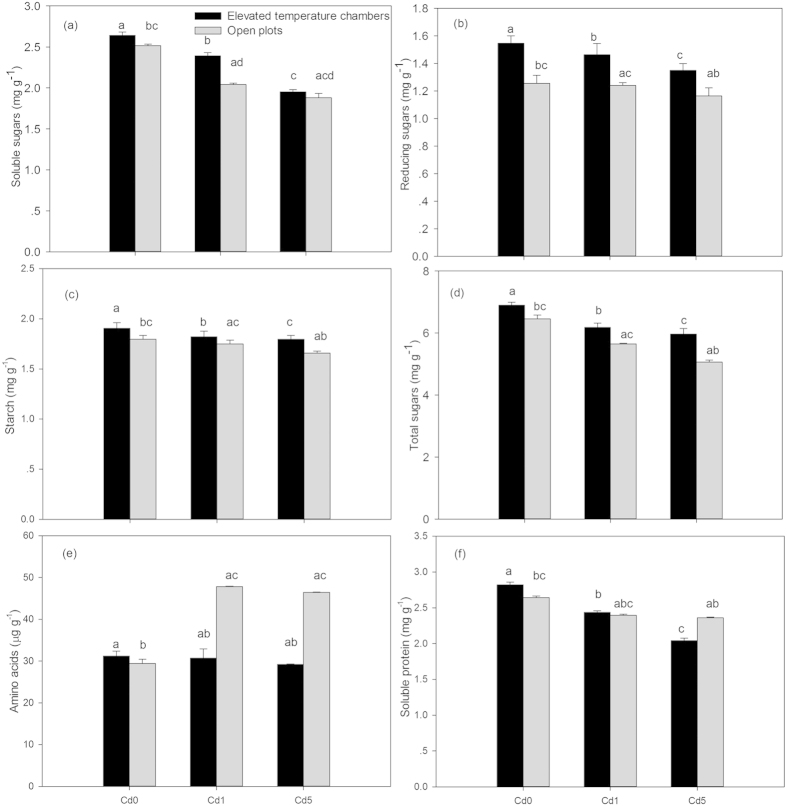
Levels of soluble sugars (a), reducing sugars (b), starch (c), total sugars (d), amino acids (e), and soluble protein (f) in tissues of wheat seedlings in elevated temperature chambers and open plots with different Cd levels. Data are means ± SE; (n = 9). Cd0, Cd1, and Cd5 in figures represented 0.3, 1.3, and 5.3 mg Cd kg^−1^ dry soil weight, respectively. Different small letters in figures represent significant differences between elevated temperature chambers and open plots, between different Cd levels under elevated temperature, and between different Cd levels in open plots at *p* < 0.05.

Treatment with elevated atmospheric temperature alone resulted in significantly increased levels of soluble sugars, reducing sugars, starch, total sugars, amino acids, and soluble protein compared to the control (Fig. 2, Table 2). Under three Cd levels (Cd0, Cd1, and Cd5), amino acids increased, while soluble sugars, reducing sugars, starch, total sugars, and soluble protein decreased, when compared to the control (Fig. 2). Soluble sugars, reducing sugars, total sugars, starch, and soluble protein showed a decreasing trend with increasing Cd under Cd stress alone (Fig. 2a–d,f).

Under three Cd levels (Cd0, Cd1, and Cd5), soluble sugars, reducing sugars, starch, and total sugars contents were enhanced by elevated temperature compared with those under Cd stress alone, which were lower under the combined treatments than under elevated temperature alone (Fig. 2a–d). In addition, soluble sugars, reducing sugars, starch, and total sugars contents showed a parallel trend to an increase in Cd levels under the combined treatments (Fig. 2a–d). Alternatively, under elevated temperature combined with both Cd1 and Cd5, amino acids content decreased by 35.8% and 37.1%, respectively, when compared to the open plots under Cd treatments (Cd1 and Cd5) and also decreased gradually with increasing Cd (Fig. 2e). Compared to Cd stress alone, soluble protein increased by 1.7% under elevated temperature + Cd1, but decreased by 13.5% under elevated temperature + Cd5 (Fig. 2f). The interaction between elevated temperature and Cd on the above photosynthetic products was significant (Table 2).

### Concentration of organic compounds in rhizosphere soil

**Figure 3:**
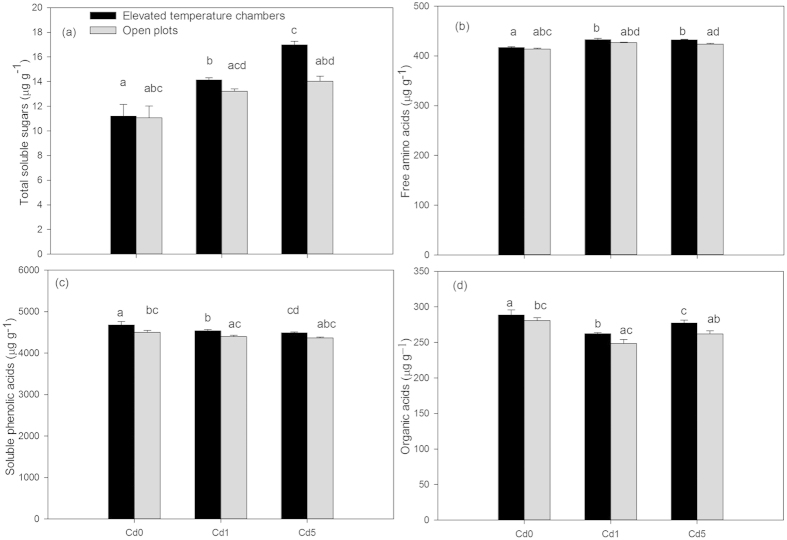
Concentrations of total soluble sugars (a), total free amino acids (b), total soluble phenolic acids (c), and organic acids (d) in rhizosphere soil in elevated temperature chambers and open plots with different Cd levels. Data are means ± SE; (n = 9). Cd0, Cd1, and Cd5 in figures represented 0.3, 1.3, and 5.3 Cd kg^−1^ dry soil weight, respectively. Different small letters in figures represent significant differences between elevated temperature chambers and open plots, between different Cd levels under elevated temperature, and between different Cd levels in open plots at *p* < 0.05.

Elevated temperature alone significantly increased the contents of total soluble sugars, free amino acids, soluble phenolic acids, and organic acids in rhizosphere soil by 1.2%, 0.8%, 4.0%, and 2.7%, respectively, compared to the control (Fig. 3). Under different Cd levels, total soluble sugars and free amino acids contents increased, while soluble phenolic acids and organic acids decreased, compared to the control. Additionally, with an increase of Cd contamination, the total soluble sugars increased gradually while soluble phenolic acids content decreased gradually.

Total soluble sugars, free amino acids, soluble phenolic acids, and organic acids were increased by elevated temperature as compared to the open plots under all three Cd treatments (Cd0, Cd1, and Cd5) (Fig. 3). Under the combined treatment, the increasing rate of total soluble sugars, free amino acids, and organic acids increased and the increasing rate of soluble phenolic acids decreased with increasing Cd levels. The interaction between elevated temperature and Cd on organic compounds in rhizosphere soil was significant with the exception of free amino acids (Table 2).

### Soil properties of the rhizosphere

**Figure 4:**
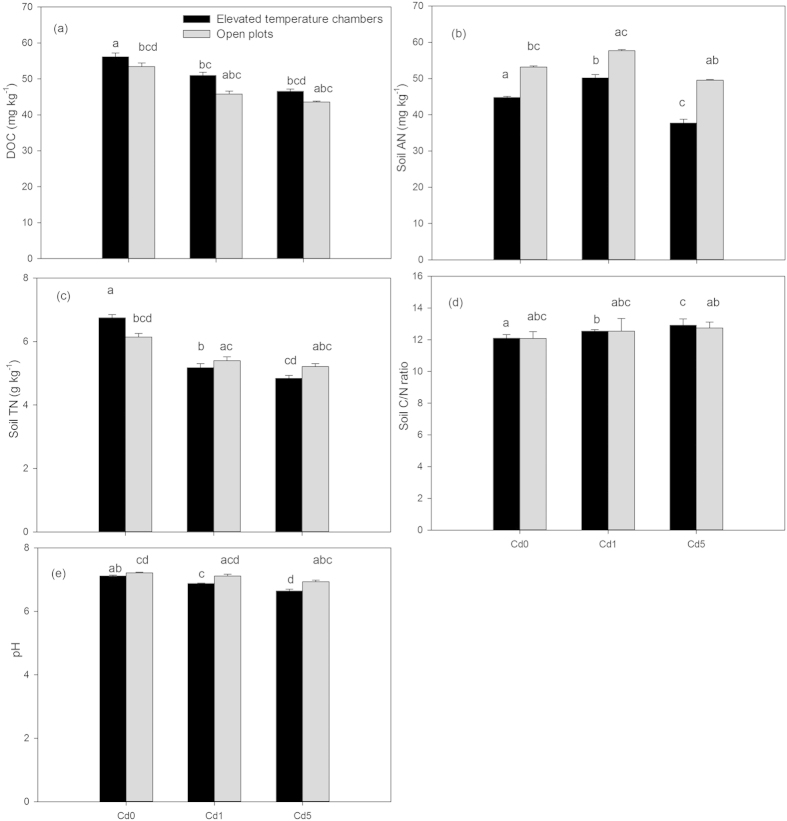
DOC (a), soil AN (b), soil TN (c), C/N ratio (d), and pH (e) in rhizosphere soil in elevated temperature chambers and open plots with different Cd levels. Data are means ± SE; (n = 9). Cd0, Cd1, and Cd5 in figures represented 0.3, 1.3, and 5.3 mg Cd kg^−1^ dry soil weight, respectively. Different small letters in figures represent significant differences between elevated temperature chambers and open plots, between different Cd levels under elevated temperature, and between different Cd levels in open plots at *p* < 0.05.

Effects of elevated temperature on DOC, soil TN (total nitrogen), soil AN (available nitrogen), C:N ratio, and pH values in rhizosphere soil were significant (Table 2). Under elevated temperature, DOC and soil TN concentrations in rhizosphere soil increased by 3.9% and 9.8%, respectively, while soil AN content decreased by 15.8% when compared to the control (Fig. 4a–c). Additionally, the C:N ratio increased by 0.05% (Fig. 4d). DOC and soil TN concentrations in rhizosphere soil decreased under different Cd levels compared to the control and showed a decreased trend with increasing Cd levels (Fig. 4a,b). In addition, the C:N ratio increased under different Cd levels, and soil AN concentrations at Cd1 and Cd5 changed by 8.5% and −6.8%, respectively, compared to the control (Fig. 4c,d).

Under Cd0, Cd1, and Cd3 levels, DOC under elevated temperature increased by 5.0%, 11.3%, and 6.8%, respectively; while C:N ratio decreased by 1.4%, 3.2%, and 4.2%, respectively, soil AN decreased by 15.8%, 13.0%, and 23.8%, respectively, and soil TN changed by 9.8%, −4.2%, and −7.2%, respectively, when compared to open plots (Fig. 4a–d). Moreover, C:N ratio was reduced by elevated temperature with increasing Cd levels. The interaction between elevated temperature and Cd on them was significant with the exception of the C:N ratio (Table 2).

Compared to the control, the pH of rhizosphere soil decreased significantly by 0.1 units under elevated temperature (Fig. 4e). Under different Cd levels, the pH decreased when compared to the control (Fig. 4e). Under three Cd levels (Cd0, Cd1, and Cd5), rhizosphere soil pH under elevated temperature decreased by 1.3%, 3.2%, and 4.2%, respectively, when compared to open plots (Fig. 4e), which indicated that rhizosphere pH value was reduced by elevated temperature with increasing Cd levels. In addition, the interaction between elevated temperature and Cd on pH was significant (Table 2).

### Enzyme activity in rhizosphere soil

**Figure 5:**
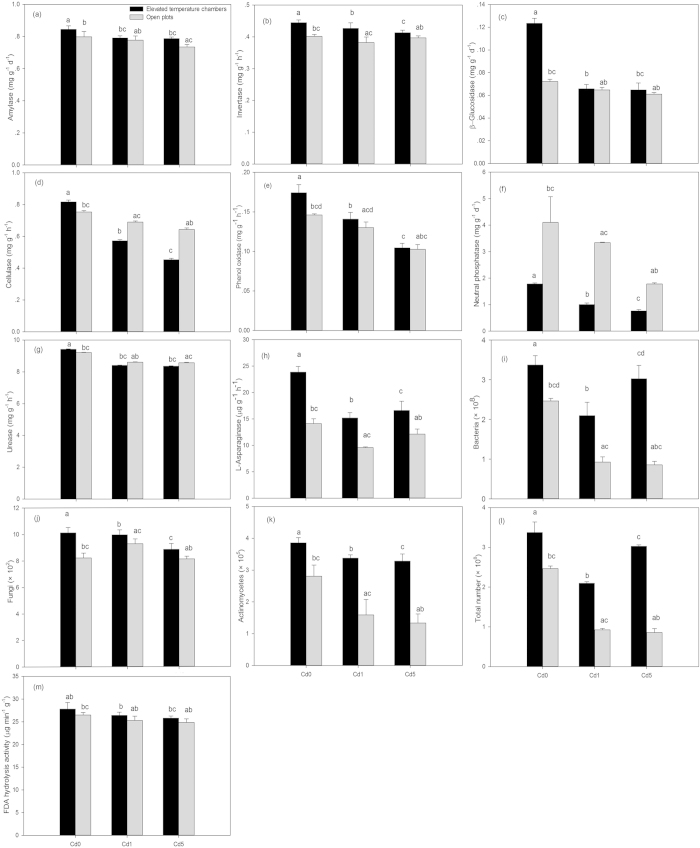
Activities of amylase (a), invertase (b), β-glucosidase (c), cellulase (d), phenol oxidase (e), neutral phosphatase (f), urease (g), and l-asparaginase (h), abundance of bacteria (i), fungi (j), actinomycetes (k), and total microorganisms (l), and FDA hydrolysis activity (m) in elevated temperature chambers and open plots with different Cd levels. Data are means ± SE; (n = 9). Cd0, Cd1, and Cd5 in figures represented 0.3, 1.3, and 5.3 Cd kg^−1^ dry soil weight, respectively. Different small letters in figures represent significant differences between elevated temperature chambers and open plots, between different Cd levels under elevated temperature, and between different Cd levels in open plots at *p* < 0.05.

Under elevated atmospheric temperature alone, the activity of amylase, invertase, β-glucosidase, cellulase, phenol oxidase, urease, and l-asparaginase increased significantly with the exception of neutral phosphatase when compared to the control (Fig. 5a-h). Under Cd stress alone, the activity of amylase, invertase, β-glucosidase, cellulase, phenol oxidase, neutral phosphatase, and urease in rhizosphere soil decreased with increasing Cd levels, and l-asparaginase under Cd1 and Cd5 levels decreased by 32.2% ang 14.1%, respectively, when compared to the control (Fig. 5a-h).

Under elevated atmospheric temperature + Cd stress, the activity of amylase, invertase, β-glucosidase, phenol oxidase, and l-asparaginase increased, but the activity of cellulase, neutral phosphatase, and urease decreased relative to Cd stress alone (Fig. 5a-h). Moreover, the increasing rate of invertase, β-glucosidase, phenol oxidase, and l-asparaginase under the combined treatments decreased with increasing Cd levels. The interaction between elevated temperature and Cd on them was significant with the exception of the invertase (Table 2).

### Abundance of culturable microorganisms and FDA (fluorescein diacetate) hydrolysis activity

Elevated temperature alone showed an increase in bacteria, fungi, actinomycetes, and the total microorganisms abundance in rhizosphere soil by 36.8%, 18.8%, 37.6%, and 36.8%, respectively, when compared to the control (Fig. 5i-f). Compared to the control, bacteria, actinomycetes, and total microorganisms abundance under different Cd levels decreased,while fungi abundance increased under Cd1 and decreased under Cd5, moreover, bacteria, actinomycetes, and total microorganisms abundance decreased with increasing Cd levels (Fig. 5i-f). Elevated temperature alone significantly improved FDA hydrolysis activity by 5.0%, while FDA hydrolysis activity decreased with increasing Cd levels (Fig. 5m, Table 2).

Under the combined treatment, bacteria, fungi, actinomycetes, and total microorganisms abundance significantly increased in comparison to Cd stress (Fig. 5i-f). Moreover, the increasing rate of bacteria, actinomycetes, and total microorganisms abundance increased with increasing Cd levels. An interaction between elevated temperature and Cd on bacteria, fungi, actinomycetes, and total microorganisms was significant (Table 2). FDA hydrolysis activity was lower under the combined treatment than under elevated temperature and higher than under Cd stress alone, moreover, it was decreased under the combined treatments with increasing Cd levels. The interaction between temperature and Cd on it was significant (Fig. 5m, Table 2).

## Discussion

Although the temperature in OTCs was in the optimal range for wheat seedlings rather than a heat shock during the first two weeks of the experiment, air temperature measured in OTCs during the third week was beyond the optimal temperature of wheat growth. Since light exposure, soil moisture, and microhabitat characteristics in both OTCs and open plots were similar, there had little effects on the growth of wheat seedlings and soil biology activity. Therefore, the effect of slightly elevated temperature was believed to be a major contributing factor to variables determined in this study.

Slightly elevated atmospheric temperature and Cd stress exhibited opposite effects on photosynthetic products in wheat seedlings. When solely taking into account elevated temperature, an increase in soluble sugars, reducing sugars, starch, total sugars, amino acids, and soluble protein showed that slightly elevated temperature in the spring stimulated the photosynthetic activity of wheat seedlings. Accumulation of Cd in wheat seedlings increased with increasing Cd levels in the study, which caused the decrease of photosynthetic product content under Cd stress alone except for amino acids, suggesting accumulation of Cd in plants could inhibit photosynthesis. Although the accumulation of Cd in wheat seedlings increased under the combined treatments compared to the Cd stress alone (Table 1), slightly elevated temperature improved the levels of soluble sugars, reducing sugars, starch, and total sugars in wheat seedlings under Cd stress. This result indicated that the stimulation of slightly elevated temperature on sugars and starch products in wheat seedlings was greater than the inhibition of Cd; However, the decrease in amino acids and soluble protein suggested that the effects of elevated temperature combined with Cd stress on photosynthetic products were still complicated. In addition, the decreased soil AN and the increased soil C/N ratio in the rhizosphere was probably contributed to the decreased amino acids and soluble protein under the combined treatments compared to Cd stress alone^24^. In conclusion, the response of reducing sugars, soluble sugars, starch, and total sugars to the combined treatment suggests that slightly elevated atmospheric temperature in the spring could improve the carbohydrate levels in wheat seedlings under Cd stress.

Organic compounds in soil are derived from the root exudates, root residues, microbial metabolism, and above ground litter^25^,^26^. Aboveground litter and weeds were removed during the experiment, which could not have contributed to soil organic compounds in this study. Therefore, total soluble sugars, free amino acids, soluble phenolic acids, and organic acids in rhizosphere soil were mainly derived from root exudates of wheat seedlings and microbial metabolism. Because a significant portion of net primary production is allocated to root systems, resulting in large fluxes of organic compounds into the soil^27^, the increased levels of photosynthetic products in wheat seedlings indirectly indicated that organic compounds in rhizosphere soil could be improved by slightly elevated temperature. In addition, the enhancements of FDA hydrolysis activity and microorganism abundance by elevated temperature could stimulate microbial metabolism of soil humus in rhizosphere soil and the fine roots of wheat seedlings, which might also contribute to the increase in organic compounds in rhizosphere soil under slightly elevated temperature^26^. Although soluble sugars in wheat seedlings decreased under Cd stress (Fig. 2a), the concentration of this compound in rhizosphere soil increased (Fig. 3a), which suggested that soluble sugars in rhizosphere soil had little relationship to the level of soluble sugars in wheat seedlings under Cd stress alone. With the exception of the Cd uptake by wheat seedlings, the decreased available Cd in rhizosphere soil at harvest compared to the soil before sowing could be also caused by the soluble phenolic acids and organic acids in root exudates that can bind metal ions such as Cd^2+^ ^28^, which resulted in the decrease of the concentrations of these compounds in rhizosphere soil that are under Cd stress alone (Fig. 3c,d). Because increased carbohydrates in wheat seedlings under elevated temperature + Cd stress (Fig. 2a–d) could result in a large flux of root exudates into rhizosphere soil^27^, it was reasonable that the total soluble sugars content in rhizosphere soil significantly increased under the combined treatments. Additionally, it is likely that increased carbohydrates in wheat seedlings (Fig. 2a–d) under the combined treatment would provide lots of carbon needed for synthesis of secondary metabolites^29^, thus increasing root exudates such as soluble sugars, organic acids, phenolic acids, and amino acids. Consequently, an increase of these components in root exudates could result in the improvement of organic compounds in rhizosphere soil under the combined treatments compared to Cd stress alone (Fig. 3). In conclusion, elevated atmospheric temperature and Cd stress exhibited opposite effects on soluble phenolic acids and organic acids, but they exhibited positive effects on total soluble sugars and free amino acids in rhizosphere soil.

Soil enzyme activity is well understood to be related to temperature, pH value, substrate concentration, and enzyme concentration; and alterations in soil enzyme activity are also related to soil properties such as pH, organic matter, and fluctuation in microbial populations^30^. Under elevated temperature alone, increased DOC, decreased soil AN, lower pH, and organic compound concentrations in rhizosphere soil (Fig. 4a,c and e and Fig. 3) provided a moderate pH value and higher substrate concentrations for enzyme kinetics. Furthermore, the increase in bacteria, fungi, actinomycetes, and total microorganisms abundance (Fig. 5i–l) was also beneficial to the improvement of soil enzyme activity in the rhizosphere. Cd inactivates enzymes by reacting with the sulfydryl groups in enzymes to form metal sulfides^31^, therefore, the increased available Cd in rhizosphere soil with increasing Cd levels in this study (Table 1) could result in the decreased activity of amylase, invertase, β-glucosidase, cellulase, phenol oxidase, neutral phosphatase, urease, and l-asparaginase under Cd stress (Fig. 5a–h). In addition, the decreased bacteria, fungi, actinomycetes, and total microorganisms abundance under different Cd levels produced less enzymes, which also resulted in the decreased enzyme activity in rhizosphere soil. Under the combined treatments compared to Cd stress alone, a less available Cd in rhizosphere soil contributed to an increase in the activity of amylase, invertase, β-glucosidase, phenol oxidase, urease, and l-asparaginase. However, the effects of both elevated temperature and soil Cd pollution on enzyme activities are complex, and the response of different enzymes to the combined treatments may vary greatly. Thus, it might lead to the decrease of cellulase, neutral phosphatase, and urease activities in rhizosphere soil under the combined treatments compared with Cd stress alone. To summarize, slightly elevated atmospheric temperature in the spring stimulated the activity of amylase, invertase, β-glucosidase, phenol oxidase, and l-asparaginase in rhizosphere soil of wheat seedlings under Cd stress. Under Cd stress alone, decreased bacteria, actinomycetes, and total microorganisms abundance and FDA hydrolysis activity were related to the increase in available Cd in rhizosphere soil with increasing Cd levels (Table 1). However, the increase in fungi abundance at Cd1 level suggested that it was more tolerant to Cd than bacteria and actinomycetes, which was consistent with the previous study^32^. Compared to Cd stress, high accumulation of organic compounds in rhizosphere soil under the combined treatment (Fig. 4) could result in an increase in bacteria, fungi, actinomycetes, and total microorganisms abundance and FDA hydrolysis (Fig. 5i–m). The direct effect of Cd stress could serve as an explanation for lower bacteria, fungi, actinomycetes, and total microorganisms abundance and FDA hydrolysis activity under the combined treatments than under elevated temperature. In addition, increased soil temperature by 1.1 °C in this study was probably within the range of daily/seasonal fluctuations. Furthermore, temperature optima of heterotrophic soil populations can be considered sufficiently broad to tolerate such limited changes in soil temperature^33^; therefore, increased soil temperature by 1.1 °C had limited impacts on soil biological activity in the rhizosphere of wheat seedlings compared to root exudates. To summarize, changes in soil biological activity in the rhizosphere of wheat seedlings under the combined treatment resulted from a number of complex effects of available Cd, soil pH, DOC, soil TN, soil AN, C:N ratio, and organic compound concentrations in rhizosphere soil.

In conclusion, slightly elevated atmospheric temperature in the spring improved the carbohydrate levels in wheat seedlings and soil organic compounds and biological activity in the rhizosphere under Cd stress in the short term. In addition, slightly elevated temperature affected available Cd by altering pH, DOC, soluble phenolic acids, and organic acids in rhizosphere soil. Slightly elevated atmospheric temperature in the spring also improved total Cd uptake by wheat seedlings, which suggested that slightly elevated temperature might interfere with efficiency of phytoremediation.

## Methods

### Plant species and soil preparation

Seeds of the common wheat *Triticum aestivum* L (Yongliang #15) were obtained from the institute of Wheat Breeding in Yongning Country, Ningxia Province, China. The Experimental soil was collected from the surface layer (0–20 cm) in a wheat field in central Shaanxi Province, China (34°16′N, 108°54′E). The soil type and chemical characteristics are shown in Table 3. Four soil concentrations of Cd (0, 1, and 5 mg kg^−1^ dry soil weight) were selected according to environmental quality standards (GB 15168-1995) and current levels of Cd pollution observed in farmland in China^34^. Fresh soil was passed through a 5 mm sieve, and the soil was artificially contaminated using CdCl_2_·2 H_2_O solution to obtain the different concentrations of 0.3 (Cd0, the control, no Cd spiked to soil), 1.3 (Cd1), and 5.3 (Cd5) mg Cd kg^−1^ soil dry weight. The artificially contaminated soils were then incubated for 30 days.

### Experimental site and design

The experimental site was located in the Weishui Campus of Chang’an University in Xi’an, China. The study site was 402 meters above sea-level and the mean annual temperature and precipitation is 13.6 °C and 508–720 mm respectively. In the experiment, we utilized an open-top chamber (OTC) facility as a passive warming device to generate an artificially warmed environment^35^. The treatment parameters were either within the OTC facility (combined Cd-contamination with elevated temperature) or in an open plot (Cd-contamination alone with ambient temperature). Each parameter was replicated three times using a randomized complete block design. Three hexagonal open-top chambers (4.4 m W × 1.6 m H) were established in the experimental garden. The distance between OTCs and the open plots was between 4 and 5 m, and the distance between the replicate blocks ranged from about 3 to 4 m. Light exposure and microhabitat characteristics in both OTCs and open plots were similar, which had little effects on the growth of wheat seedlings. Automated measurements of temperature, humidity, and soil water content were taken every minute of every day throughout the experiment. The air temperature at 20 cm aboveground the pots, soil temperature at 5 cm depth in the pots, and the soil water content at a 5 cm depth in the pots within each chamber or open plot were recorded in ten minute intervals. The air temperature (at 20 cm aboveground the pots) and soil temperature (at 5 cm depth) in the pots within the chambers increased by an average of 2.6 °C and 1.1 °C, respectively, compared to the open plots. To guarantee comparable non-limiting soil moisture conditions in all pots, wheat seedlings were watered by monitoring with a hand-held probe (IMKO, Germany) every 4 days and the pots were maintained at 60% field capacity during the seedling growth.

### Design of the pot experiment

The experiment was performed in 45 cm D × 50 cm H plastic pots. Seven root bags were placed in each pot. Each root bag was filled with 800 g Cd-contaminated soil and covered with 19.4 kg soil. Each Cd-contamination parameter was prepared in triplicate. The pots were placed both in the OTCs and open plots. In 2013 April, *T. aestivum* seeds were planted in all of the pots to yield 91 seedlings per pot after emergence. Litter and weeds were monitored and removed from pots by hand to reduce effects of them on variables measured in this experiment. The three treatment parameters were: (1) the control (open plots with Cd0), (2) Cd-contaminated soils (open plots + Cd1, and Cd5), (3) elevated temperature chambers and Cd-contaminated soils (elevated temperature chambers + Cd1 and Cd5), and (4) elevated temperature only (elevated temperature chambers + Cd0). The influence of elevated temperature was examined by comparing (4) with (1). The effect of Cd stress alone was investigated by comparing (2) with (1). The effect of elevated temperature on photosynthetic products in wheat seedlings and on organic compounds and biological activity in rhizosphere soil under Cd stress was assessed by comparing (3) with (2).

### Rhizosphere soil sampling

Soil from the rhizosphere of 3-week-old wheat seedling was used for analysis of soil properties, organic compounds, and enzyme and microbial activity. Rhizosphere soil was defined as the soil that was strongly adhered to roots in the root bags or within the space penetrated by roots in the root bags^36^. Roots were retrieved from the root bags, and the rhizosphere soil was collected and mixed. The rhizosphere soil samples were divided into three parts: one soil sample was stored at 4 °C for analysis of enzyme activity, cultivable microorganism population, microbial biomass, and FDA (fluorescein diacetate) hydrolysis activity, the second soil sample was stored at −20 °C for the analysis of sugars, phenolic, organic, and amino acid concentrations, and the third soil sample was air-dried for analysis of soil pH, DOC, soil TN, and soil AN.

### Analysis of photosynthetic products in wheat seedlings

Wheat seedlings (3 weeks old) were harvested, separated from roots, and immediately immersed in liquid nitrogen, for analysis of photosynthetic products. Soluble carbohydrates were extracted by the method of Rogers *et al.*^37^. Total sugars and reduced sugars contents were measured by levels of phenol-sulphuric acid^38^. Soluble sugars content was determined as depicted by Dubois *et al.*^38^, and the content of starch was determined by the glucose oxidase-peroxidase (GOD-POD)/2-2′-azino-bis (3-ehtylbenzthiazoline-6-sulfonic -c acid) (ABTS) assay using glucose as standard^39^. Soluble protein content was determined by following the method of Bradford^40^. Amino acids were evaluated using the ninhydrin colorimetric method^41^.

### Analysis of organic compounds in the rhizosphere soil

Total soluble sugars were measured by the phenol-sulfuric acid colorimetric procedure^38^. Free amino acids were analyzed using the ninhydrin colorimetric method^39^. Soluble phenolic acids were evaluated using the Folin-Ciocalteu method as detailed in Deforest *et al.*^42^. Organic acids were determined by spectrophotometry with FeCl_3_ using acetic acid as a standard^43^. Organic acids were extracted from 10 g of 2 mm sieved soil using 40 mL of purified water for 1 h and then centrifuged at 10,000 × g for 10 min. 10 mL of the supernatant mixed with 0.25 mL of 0.2 mol L^−1^ NaOH was dried by Termovap Sample Concentrator, then 2.5 mL of acid ethylene glycol (ethylene glycol: sulfuric acid = 47:3) was added to it and with 80 °C water bath for 8 min in order to dissolve salt. After cooled by cold bath, 1 mL of 10% (m/v) hydroxylamine hydrochloride and 4 mL of 4.5 mol L^−1^ NaOH were added to the volumetric flasks and then were homogenized. After 3 min, 12 mL of 0.15 mol L^−1^ FeCl_3_ was added to the volumetric flasks and dilute to a final volume of 50 mL using purified water. After 20 min, the absorbance of the solution was read at 500 nm.

### Analysis of enzyme activity, abundance of culturable microorganisms, and microbial activity in the rhizosphere

Eight enzymatic activities were measured as described below. The activity of phenol oxidase was determined using l–3, 4-dihydroxyphenylalanine (L-DOPA) as a substrate (expressed as μg DOPA converted h^−1^ g^−1^ dry soil equivalent^−1^)^44^. Amylase activity was measured by starch hydrolysis (expressed as μg maltose d^−1^ g^−1^ dry soil equivalent^−1^)^45^. Soil β-glucosidase activity was determined using spectrophotometric assays by incubating one gram of the air-dried soil for one hour with p-nitrophenyl-β-D-glucoside at a pH of 6.0 (expressed as μg p-nitrophenol d^−1^ g^−1^ dry soil equivalent^−1^)^46^. According to the methods of Xu and Zheng^47^, invertase was determined by incubating 5 g soil with 15 mL of 8% (m/v) sucrose for 24 h at 37  °C. The suspension reacted with DNS (3,5-dinitrosalicylic acid) for the colorimertric assay and the absorbance was read at 508 nm (reported as μg glucose h^−1^ g^−1^ dry soil equivalent^−1^). Cellulase enzymatic activity was calculated by the hydrolysis of carboxmethyl-cellulose (CMC) using the methodology specified by Pancholy and Rice (reported as μg glucose d^−1^ g^−1^ dry soil equivalent^−1^)^48^. Urease activity was approximated as the amount of NH_4_^+^ released in the hydrolysis of urea as a substrate in Tris buffer (reported as mg NH_4_^+^-N h^−1^ g^−1^ dry soil equivalent^−1^)^49^. l-asparaginase –asparaginase activity was evaluated using the methodology given by Frankenberger and Tabatabai^50^ (expressed as μg ammonia h^−1^ g^−1^ dry soil equivalent^−1^). Neutral phosphatase activity was determined spectrophotometrically by the disodium phenyl phosphate method employed by Wu^51^ (reported as μg phenol d^−1^ g^−1^ dry soil equivalent^−1^). Flourescein diacetate FDA hydrolysis activity was determined as indicated by Mora *et al.*^52^. Enzymatic activity (U) was expressed as the quantity of fluorescein released (expressed as μg fluorescein min^−1^ g^−1^ dry soil equivalent^−1^). All enzymatic results were expressed on a dry weight basis, and each enzyme activity in each chamber and open plot were done in triplicate. Colony-forming units (CFUs) of bacteria, actinomycetes, and fungal were determined using a modified plate-dilution technique on meat peptone agar, Gause’s starch agar, and Thayer-Martin agar, respectively^53^.

### Soil properties

The pH of the rhizosphere soil under each different treatment parameter was measured by a pH meter after adding soil to distilled water without CO_2_ at a 1:2.5 ratio (w/v)^54^. The K_2_Cr_2_O_7_–H_2_SO_4_ oxidation method was used to measure the TOC content in the soil^55^. The content of soil DOC was measured by adding soil to water at a ratio of 1:10 (w/v) and analyzed using the automated Shimazu, TOC-500 TOC analyzer. Soil TN content was analyzed using the Kjeldahl method^56^. Calculation of the soil carbon-to-nitrogen (C:N) ratio was based on the total content of both carbon and nitrogen. Soil AN concentration was determined by persulfate digestion^57^.

### Concentration of available Cd in the soil before sowing and in th rhizosphere soil at harvest

20 mL Mehlich-III solution (0.2 mol L^−1^ CH_3_COOH + 0.25 mol L^−1^ NH_4_NO_3_ + 0.015 mol L^−1^ NH_4_F + 0.015 mol L^−1^ HNO_3_ + 0.001 mol L^−1^ EDTA at pH 2.5) was added to 5 g of soil dried at room temperature before sowing and at harvest and the suspension was stirred for 5 min^58^. Then the suspension was filtered through 0.45 μm nylon filters. The filtrate was used for the determination of available Cd using an Atomic Absorption Spectrometer (AAS) and a graphite tube equipped with an automatioc sampler (ZEEnit 700, Analytikjena, Germany). Calibration curves were prepared based on 1000 mg L^−1^ of commercial standard solutions (Sigma). The reliability of the digestion and analytical procedure was assed using blanks and standards as quality assurance/quality control (QA/QC).

### Uptake of Cd by wheat seedlings

To determine the uptake of Cd by wheat seedlings, the whole wheat seedlings, which were washed with water to remove soil particles on roots, were dried in an oven at 75 °C for 72 h. Then wheat seedlings dried were digested in a mixture of HNO_3_ and H_2_O_2_ (8:1, v/v) using a microwave digestion method (CEM, Mars:240/50, Ameria). The Cd content in the extractants was determined using Atomic Atomic Absorption Spectrometer (AAS) and a graphite tube equipped with an automatioc sampler (ZEEnit 700, Analytikjena, Germany).

### Statistical analysis

A two-way analysis of variance (ANOVA) was used to exam the combined effect of elevated temperature and Cd stress on photosynthetic products in wheat seedlings, organic compounds in rhizophere soil, soil properties, enzyme activity, FDA hydrolysis and microbial activity, abundance of culturable microorganisms,avilable Cd in the soil before sowing and at harvest, and the uptake of Cd by wheat seedlings. One-way ANOVA was used to investigate the effects of elevated temperature or Cd stress on these parameters. The normal distribution of data was tested by Kolmogorov-Smirnov test. All statistical tests were computed with SPSS statistic software (IBM Inc, version 16.0).

## Additional Information

**How to cite this article**: Jia, X. *et al.* Elevated temperature altered photosynthetic products in wheat seedlings and organic compounds and biological activity in rhizopshere soil under cadmium stress. *Sci. Rep.* **5**, 14426; doi: 10.1038/srep14426 (2015).

## Figures and Tables

**Table 1:** Effect of elevated temperature on available Cd in the soil before sowing and in rhizosphere soil at harvest and total Cd uptake by wheat seedlings. Values are the mean + SE (n = 3).

Cd levels	Temperature	Available Cd (mg kg^−1^ dry weight soil)		Cd uptake (mg kg^−1^ dry plants)
		Before sowing	At harvest	
Cd0	Open plots	0.18 ± 0.01	0.08 ± 0.01**	0.58 ± 0.03A
	Elevated temperature		0.06 ± 0.01**	0.67 ± 0.01B
Cd1	Open plots	0.47 ± 0.02	0.35 ± 0.03**a	3.55 ± 0.13A
	Elevated temperature		0.24 ± 0.04**b	4.36 ± 0.17B
Cd5	Open plots	1.59 ± 0.13	1.31 ± 0.08*A	14.66 ± 1.03A
	Elevated temperature		1.01 ± 0.03**B	18.83 ± 1.10B

a and b represent the significant difference between open plots and elevated temperature at *p* < 0.05. A and B represent the significant difference between open plots and elevated temperature at *p* < 0.01. ** and * represent the significant difference between before sowing and at harvest (^*^p < 0.05; ^**^p < 0.01).

**Table 2:** Results of two-way ANOVA (F value) examining effects of elevated temperature and Cd pollution on primary metabolites levels in tissues and soil organic compounds and soil biological activities in the rhizosphere of wheat seedlings.

Variables		Elevated temperature	Cd	Elevated temperature × Cd
Photosynthesis products	Reducing sugar	(25.8)**	(22.4)**	(83.0)**
	Soluble sugar	(163.7)**	(29.2)**	(4.2)*
	Starch	(45.7)**	(113.4)**	(11.6)**
	Total sugar	(24.4)**	(11.1)**	(7.1)**
	Amino acid	(9.5)**	(36.2)**	(12.2)**
	Soluble protein	(322.4)**	(3.6)***	(74.4)**
Soil properties	DOC	(91.6)**	(34.8)**	(25.6)**
	Soil TN	(73.3)**	(1.1)Ns	(5.6)**
	Soil AN	(156.1)**	(379.2)**	(7.6)**
	C/N ration	(20.5)**	(0.9)Ns	(2.7)Ns
	pH	(122.6)**	(136.9)**	(10.9)**
Organic compounds in rhizosphere soil	Soluble sugars	(75.1)**	(19.4)**	(8.5)**
	Free amino acids	(39.5)**	(16.6)**	(1.1)Ns
	Phenolic acids	(43.2)**	(4.7)**	(19.3)**
	Organic acids	(237.6)**	(123.8)**	(46.8)**
Enzyme activities	Amylase	(10.5)**	(5.4)**	(6.2)**
	Invertase	(26.2)**	(5.7)**	(3.5)Ns
	β-Glucosidase	(7.1)**	(10.0)**	(34.9)**
	Cellulase	(48.3)**	(147.5)**	(15.3)**
	Phenol oxidase	(48.6)**	(16.2)**	(41.3)**
	Neutral phosphatase	(5.6)**	(35.1)**	(36.5)**
	Urease	(20.0)**	(7.2)**	(4.8)**
	l-Asparaginase	(43.4)**	(74.3)**	(6.9)**
Microbial abundance and activities	Bacteria	(10.9)**	(43.7)**	(281.8)**
	Fungi	(75.5)**	(72.4)**	(80.7)**
	Actinomycetes	(10.1)**	(11.5)**	(26.8)**
	Total microorganisms	(27.0)**	(222.7)**	(5.8)**
	FDA hydrolysis activity	(142.1)**	(88.0)**	(6.4)**

Ns, not significant; ^*^ *p* < 0.05; ^**^ *p* < 0.01.

**Table 3:** Type and chemical characteristics of the used soil.

Soil type	Leached brown soil (according to Chinese soil classification)
pH	8.4
Organic matter content (g kg^−1^)	11.4 ± 1.0
Organic carbon (g kg^−1^)	6.7 ± 0.1
Total nitrogen (g kg^−1^)	1.1 ± 0.1
Soluble salts (mg kg^−1^)	383.5 ± 1.0
Available N (mg kg^−1^)	0.1 ± 0.0
Available P (mg kg^−1^)	4.4 ± 0.4
Available K (mg kg^−1^)	133.4 ± 0. 9
Cation exchange capacity (meq 100 g^−1^)	26.4 ± 1.1
Total Cd (mg kg^−1^)	0.3 ± 0.02
